# Dose of Alcohol From Beer Required for Acute Reduction in Arterial Stiffness

**DOI:** 10.3389/fphys.2020.01033

**Published:** 2020-08-28

**Authors:** Masato Nishiwaki, Takahiro Yamaguchi, Ren Nishida, Naoyuki Matsumoto

**Affiliations:** ^1^Faculty of Engineering, Osaka Institute of Technology, Osaka, Japan; ^2^Faculty of Environmental & Symbiotic Sciences, Prefectural University of Kumamoto, Kumamoto, Japan

**Keywords:** alcohol, arteriosclerosis, blood pressure, cardio-ankle vascular index, pulse wave velocity

## Abstract

Acute beer or alcohol ingestion reduces arterial stiffness, but the dose required to reduce arterial stiffness is unclear. Therefore, this study aimed to determine the acute effects of ingesting various amounts of beer on arterial stiffness in healthy men. Nine men (20–22 years) participated, in eight trials in random order on different days. The participants each consumed 25, 50, 100, or 200 mL of alcohol-free beer (AFB25, AFB50, AFB100, and AFB200) or regular beer (B25, B50, B100, and B200), and were monitored for 60 min thereafter. Arterial stiffness did not significantly change among all AFB and B25. However, B50, B100, and B200 caused a significant decrease in arterial stiffness for approximately 30–60 min: heart-brachial pulse wave velocity (B50: −4.5 ± 2.4%; B100: −3.4 ± 1.3%; B200: −8.1 ± 2.6%); brachial-ankle pulse wave velocity (B50: −0.6 ± 2.0%; B100: −3.3 ± 1.1%; B200: −9.3 ± 3.0%); heart-ankle pulse wave velocity (B50: −3.7 ± 0.3%; B100: −3.3 ± 0.9%; B200: −8.1 ± 2.7%); and cardio-ankle vascular index (B50: −4.6 ± 1.3%; B100: −5.6 ± 0.8%; B200: −10.3 ± 3.1%). Positive control alcoholic beverages reduced arterial stiffness, and these reductions did not significantly differ regardless of the type of beverage. Our data show that consuming about 50 mL of beer can start to reduce arterial stiffness, and that the reduced arterial stiffness is mainly attributable to the alcohol in beer.

## Introduction

Arterial stiffening occurs with advancing age and impairs the ability of arteries to buffer BP pulsation and blood flow (Avolio et al., 1983; Tanaka and Safar, 2005). Thus, increased arterial stiffness has been identified as an independent risk factor for future cardiovascular diseases (Laurent and Boutouyrie, 2007; Vlachopoulos et al., 2010; Tanaka, 2019). Pulse wave velocity (PWV) and cardio-ankle vascular index (CAVI) are established indices of arterial stiffness (Vlachopoulos et al., 2010; Shirai et al., 2011). In particular, large elastic arteries progressively stiffen with advancing age, even in healthy individuals (Avolio et al., 1983; Gando et al., 2010). However, physical activity and dietary habits can alter the degree of arterial stiffening associated with age (Tanaka and Safar, 2005; Gando et al., 2016; Tanaka, 2019). Therefore, further scientific evidence of simple and effective methods for preventing arterial stiffening is needed.

Epidemiological studies have identified a J-shaped association between alcohol intake and arterial stiffness, and arterial stiffness is significantly lower in those who consume light-to-moderate amounts of alcohol than in those who consume none (Sierksma et al., 2004; Hougaku et al., 2005; Mattace-Raso et al., 2005; Di Castelnuovo et al., 2006; Costanzo et al., 2011; Poli et al., 2013; Panagiotakos et al., 2019). Beer is one of the most popular alcoholic beverages worldwide, and several studies have noted the vascular protective effects of beer or alcoholic beverages (Toda and Ayajiki, 2010; Arranz et al., 2012; de Gaetano et al., 2016; Zhou et al., 2016). Indeed, acute effects of beer or alcohol ingestion have been studied extensively; 500 mL red wine (0.8 g ethanol/kg body weight) (Mahmud and Feely, 2002), red wine and water, vodka and water, or beer (0.32 g ethanol/kg body weight and 7 mL/kg body weight of total volume) (Krnic et al., 2011), 400 mL beer and 400 mL water (total 800 mL), 800 mL dealcoholized beer, or 67 mL vodka and 733 mL water (total 800 mL) (approximately 0.21 g ethanol/kg body weight) (Karatzi et al., 2013), and 250 mL red wine (approximately 0.34 g ethanol/kg body weight) (Fantin et al., 2016). The findings of our recent study also showed that ingesting 200 mL of beer (0.14 g ethanol/kg body weight), corresponding to the lower daily limit of a mild-to-moderate drinker, reduces arterial stiffness in healthy young men (Nishiwaki et al., 2017a). That is, only a small amount of beer or alcohol may reduce or prevent arterial stiffening.

In contrast, many studies have indicated that moderate-to-excessive alcohol consumption increases arterial stiffness and the risk of cardiovascular diseases (Arranz et al., 2012; de Gaetano et al., 2016; Zhou et al., 2016; Wood et al., 2018). Low concentrations of alcohol increase endothelial function in human endothelial cells through nitric oxide (NO) production, whereas high concentrations induce endothelial dysfunction and apoptosis (Toda and Ayajiki, 2010; Zhou et al., 2016). Furthermore, the J-shaped association has not gained complete acceptance, especially from the viewpoint of alcohol intake and stroke, cancer, and liver diseases (Arranz et al., 2012; de Gaetano et al., 2016; Zhou et al., 2016; Wood et al., 2018). Several findings imply that moderate-to-excessive alcohol consumption confers negative effects on vascular health or longevity (Arranz et al., 2012; Goslawski et al., 2013; de Gaetano et al., 2016; Zhou et al., 2016; Wood et al., 2018). Accordingly, habitual consumption of small amounts of beer might be important to reduce or prevent arterial stiffening and positively affect health and longevity. However, as far as we can ascertain, little is known about the required dose of beer or alcohol required to reduce arterial stiffness.

Based on this background, the present study aimed to determine how much beer should be consumed to elicit reductions in arterial stiffness. Therefore, we investigated the acute effects of ingesting various amounts of beer on arterial stiffness in healthy young men. Considering our previous results (Nishiwaki et al., 2017a), we hypothesized the presence of a critical threshold of reductions in arterial stiffness when healthy young men ingest <200 mL of beer.

## Materials and Methods

### Participants

The mean age, height, body mass, body mass index (BMI), and body fat in nine healthy young males participated in this study were 21.1 ± 0.2 years, 171.0 ± 2.1 cm, 67.3 ± 3.8 kg, 22.9 ± 1.1 kg/m^2^, and 21.4 ± 2.0%, respectively. Because elastic properties of arteries fluctuate significantly with the phases of the menstrual cycle in young female (Hayashi et al., 2006), only male participants were recruited in this study. None of them had chronic diseases that could affect cardiovascular health, metabolism, or daily physical activity, a history of smoking, or were presently under medication. They all habitually consumed beverages containing alcohol, but none exceeded the recommended amount of alcohol consumption (40 g/day for men in Japan) beyond which the risk of developing lifestyle-related diseases is increased. The purpose, procedures, and risks of the study were explained to each participant. All participants provided written informed consent before participating in the study, which was reviewed and approved by the Human Ethics Committee at the Osaka Institute of Technology (approval numbers: 2016-5 and 2017-55) and implemented in accordance with the guidelines of the Declaration of Helsinki.

### Sample Size and Experimental Procedures

We determined the sample size would be appropriate for the study by power calculations using G^∗^Power 3. In accordance with our previous findings (Nishiwaki et al., 2017a), we assumed that arterial stiffness would be transiently reduced by ∼10%. At least eight participants were needed to detect this difference at 80% power with a two-tailed α of 5%. We therefore planned to recruit more than eight participants in this study.

All experiments were conducted in a quiet, air-conditioned room at 22–24°C. To avoid potential diurnal variations, all experiments proceeded at the same time of day at least 4 h after a light meal. All participants abstained from beverages containing alcohol and caffeine, and avoided strenuous physical activity for 12 h before participating in experiments. In addition, the participants were advised to eat their habitual breakfast, lunch, and dinner on the day before each experiment.

**FIGURE 1:**
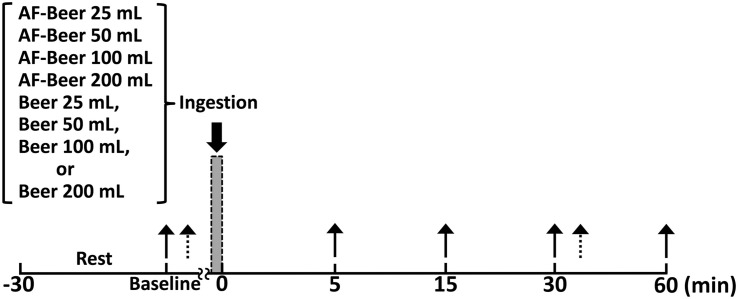
Time course of the experiment. Solid arrows: time points of measured breath alcohol level, heart-brachial pulse wave velocity, brachial-ankle pulse wave velocity, heart-ankle pulse wave velocity, cardio-ankle vascular index, and hemodynamics; broken arrows: time points of measured carotid-femoral pulse wave velocity; AF-Beer: alcohol-free beer.

Figure 1 shows the time course of the study. All participants were assigned in random sequence to one trial per day for 8 days. The trials comprised the consumption of 25 (AF25), 50 (AF50), 100 (AF100), and 200 (AF200) mL of alcohol-free beer, and the same volumes of regular beer (B25, B50, B100, and B200, respectively). That is, the amounts (mL) of beer consumed by each participant relative to body mass (kg) were 0.38 ± 0.03 mL/kg (B25), 0.77 ± 0.05 mL/kg (B50), 1.53 ± 0.10 mL/kg (B100), and 3.06 ± 0.21 mL/kg (B200). Also, the amounts (g) of alcohol consumed by each participant relative to body mass (kg) were 0.017 ± 0.001 g/kg (B25), 0.034 ± 0.002 g/kg (B50), 0.067 ± 0.005 g/kg (B100), and 0.135 ± 0.009 g/kg (B200). The participants arrived at the laboratory every day for 8 days and rested for at least 30 min, then breath alcohol concentration (BAC), heart rate (HR), blood pressure (BP), and PWV were assessed to establish pre-ingestion baselines. Participants then consumed test drinks over a period of 3 min without food. The test beer (B) containing 5.5 vol% alcohol was The Premium Malt’s (Suntory Holdings, Osaka, Japan) and the control alcohol-free (AF) beer was All Free (Suntory Holdings, Osaka, Japan). All test drinks were poured into paper cups to blind the participants to which beer they consumed (single-blind study). After consuming a test drink, each participant rested on a comfortable chair for 60 min while the biometric measurements were repeated at 5, 15, 30, and 60 min post-ingestion.

### Assessment of Each Parameter

The same investigators measured all parameters. BAC were measured in triplicate using an AL-1 breath alcohol detector (Daiji Industry, Osaka, Japan), and the mean values were analyzed (Nishiwaki et al., 2017a). The coefficients of variation (CV; a measure of reproducibility) of BAC measurement on two separate days were 9.4 ± 1.6%. According to a previous study (Nishiwaki et al., 2017a), circulating alcohol levels (%) were estimated using the formula: BAC (mg/L)/5. HR, BP, and PWV were measured using a semi-automated device (VS-1500AE/AN; Fukuda Denshi, Tokyo, Japan) with participants in the supine position (Nishiwaki et al., 2014, 2017b, 2019). Cuffs to measure BP and PWV were wrapped around both brachial upper arms and ankles, and then heart-brachial PWV (hbPWV), brachial-ankle PWV (baPWV), heart-ankle PWV (haPWV), and cardio-ankle vascular index (CAVI) were used as indexes of arterial stiffness. The carotid-femoral PWV (cfPWV), which is an index of central arterial stiffness, was measured at baseline (Before) and 30–45 min post-ingestion (After) using the same device (Nishiwaki et al., 2017a). Carotid and femoral arterial pressure waveforms were recorded by amorphous pulse wave sensors (TY-501A; Fukuda Denshi) attached to carotid and femoral arteries, and values were automatically calculated as the distance between the carotid and femoral artery sites divided by the transit time. The CVs of cfPWV, hbPWV, baPWV, haPWV, and CAVI measurements on two separate days were 7.5 ± 1.2, 4.2 ± 0.6, 2.7 ± 0.3, 2.6 ± 0.6, and 3.6 ± 0.6%, respectively (Nishiwaki et al., 2015, 2017a,2017b, 2019).

### Supplementary Experiments

We conducted a positive control study to confirm whether alcohol in beer is the main source of changes in arterial stiffness (Supplementary Experiment 1). Spirytus 96° (96 vol% alcohol) vodka (Polmos Warszawa, Warszawa, Poland) was combined with alcohol-free beer to a final concentration as 5.5 vol% (AF + Alc) to coincide with the test beer. We then investigated the effects of ingesting this mixture on arterial stiffness in five healthy young males who participated in three trials in random order on separate days as follows: 50 mL of AF (AF50), 25 mL of AF + Alc (AF + Alc25), and 50 mL of AF + Alc (AF + Alc 50).

Supplementary Experiment 2 aimed to confirm whether ingesting different types of alcoholic beverages induce similar reductions in arterial stiffness. In random order on separate days, 10 healthy young males participated in five trials, and consumed the following on separate days: 100 mL each of pure water (PW), beer (B), sake (S), Japanese distilled spirits (D), and whisky (W). Reverse osmosis water was used for the PW. S of 13–14 vol% alcohol (Thuki, Gekkeikan Sake, Kyoto, Japan), D of 25 vol% alcohol (Kurokirishima, Kirishima Shuzo, Kagoshima, Japan), and W of 40 vol% alcohol (Suntory Whisky Kakubin, Suntory Holdings, Osaka, Japan) were combined with PW, then the mixture was adjusted to 5.5 vol% alcohol to match the test beer. All Supplementary Experiments proceeded in the same manner as the main study, and BAC, systemic PWVs, and CAVI were assessed at baseline and at 5, 15, 30, and 60 min post-ingestion.

### Statistical Analysis

Results are presented as means ± SEM. Parameters at each baseline were compared using one-way repeated-measures ANOVA. Changes in each parameter were analyzed by two-way (trial × time) repeated-measures ANOVA. When the *F*-value was significant, the Bonferroni correction was applied for *post hoc* multiple comparisons. Relationships were assessed using Pearson’s correlation coefficient. All data were statistically analyzed using IBM SPSS Statistics 25J (IBM Japan, Tokyo, Japan) and Bell Curve for Excel Statistics 3.10 (Social Survey Research Information Co., Tokyo, Japan). To quantify the magnitude of the experimental effect between baseline and object value, effect size (ES) was calculated using G^∗^Power 3. Differences were considered significant at *P* < 0.05.

## Results

### Effects of Ingesting Beer on BAC and Arterial Stiffness

**FIGURE 2:**
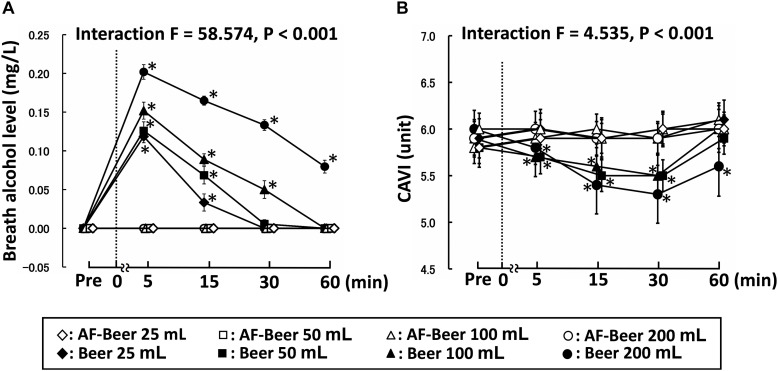
Effects of ingesting different doses of beer on alcohol level **(A)** and CAVI **(B)**. Broken lines: time point of beverage ingestion; Pre: baseline; AF-Beer: alcohol-free beer ingestion; CAVI: cardio-ankle vascular index; *p < 0.05 vs. each pre; data are expressed as mean ± SE.

Two-way repeated-measures ANOVA revealed significant interaction in BAC (Figure 2A). Baseline BAC did not significantly differ across trials. The BAC in all AF trials did not significantly change throughout the experimental period, whereas that in all the beer trials changed significantly at 5 min and 15 min post-ingestion (*P* < 0.01). In particular, the BAC at B100 increased until 30 min (*P* < 0.01) and that of B200 increased throughout the study period (*P* < 0.01).

Two-way repeated-measures ANOVA also revealed significant interactions in hbPWV, baPWV, haPWV, and CAVI (Table 1 and Figure 2B). Baseline PWV and CAVI did not differ significantly across trials as well as in all AF trials. Conversely, hbPWV (B100 15 min, ES = 0.88; B200 15 min, ES = 1.09), baPWV (B100 15 min, ES = 1.10; B200 15 min, ES = 1.06), haPWV (B100 15 min, ES = 1.25; B200 15 min, ES = 1.02), and CAVI (B100 15 min, ES = 2.04; B200 15 min, ES = 1.35) were decreased, particularly in the B100 and B200 trials at 30–60 min post-ingestion. In the B50 trial, hbPWV (15 min ES = 0.58; 30 min ES = 1.09), haPWV (15 min, ES = 4.18; 30 min, ES = 2.40), and CAVI (15 min, ES = 1.28; 30 min, ES = 3.00) were significantly reduced at about 30 min, but baPWV (15 min, ES = 0.06; 30 min, ES = 0.50) tended to decrease from each baseline level and baPWV responses were relatively blunt (small change). Neither PWV nor CAVI reduced significantly during post-ingestion of B25 (hbPWV 15 min, ES = 0.18; baPWV 15 min, ES = 0.04; haPWV 15 min, ES = 0.21; CAVI 15 min, ES = 0.00). The cfPWV did not change significantly in all AF trials. Conversely, cfPWV was significantly reduced in the B50 (ES = 0.42), B100 (ES = 0.50), and B200 (ES = 0.72) trials in a comparison of before and after values (each *P* < 0.05), but not in the B25 trial (ES = 0.39).

**TABLE 1:** Effects of ingesting different doses of beer on PWVs.

**Variables**	**Baseline**	**5 min**	**15 min**	**30 min**	**60 min**	**Trial**	**Time**	**Interaction**
Heart-brachial PWV, cm/s								
AF25	32411	3288	3299	33211	33710	*F* = 0.500	*F* = 11.521	*F* = 3.443
AF50	3209	3318*	3369*	34611*	3399*			
AF100	31711	32510*	32911*	32610*	3357*	*P* = 0.831	*P* < 0.001	*P* < 0.001
AF200	3218	33211*	3269	3409*	33612*			
B25	32211	3217	32410	3339*	34310*			
B50	3269	3218	31212*	31511*	32811			
B100	32712	33316	31612*	31314*	33614			
B200	32510	31811*	29913*	30114*	31712*			
Brachial-ankle PWV, cm/s								
AF25	107329	106920	104223	103926	105037	*F* = 0.172	*F* = 6.616	*F* = 2.066
AF50	105333	105427	105939	105226	106626			
AF100	107932	106132	108240	107126	108234	*P* = 0.990	*P* < 0.001	*P* = 0.002
AF200	106926	106627	107033	106429	105731			
B25	108228	110341	108544	108140	106839			
B50	106225	105231	105838	104030	104034			
B100	107430	106632	103931*	102933*	105042*			
B200	109436	108237	99452*	100654*	103954*			
Heart-ankle PWV, cm/s								
AF25	60613	60511	59912	60315	61016	*F* = 0.459	*F* = 7.854	*F* = 3.390
AF50	6069	60313	60917	61615	61513			
AF100	59614	59615	60816	60311	61414	*P* = 0.861	*P* < 0.001	*P* < 0.001
AF200	59912	60713	60213	61414	60714			
B25	60218	60715	60719	60418	61017			
B50	60112	59812	57912*	57914*	59513			
B100	60415	60719	58417*	57919*	60420			
B200	60417	59615	55624*	55824*	58425*			
Carotid-femoral PWV, cm/s								
AF25	61116	−	−	61318	−	*F* = 0.188	*F* = 0.772	*F* = 1.395
AF50	60618	−	−	60421	−			
AF100	62316	−	−	61816	−	*P* = 0.987	*P* = 0.383	*P* = 0.223
AF200	61321	−	−	61918	−			
B25	58917	−	−	61219*	−			
B50	62222	−	−	59316*	−			
B100	62019	−	−	60618*	−			
B200	61010	−	−	59313*	−			

Data are expressed as mean ± SE. PWV, pulse wave velocity; AF25, AF50, AF100, and AF200, alcohol-free beer of 25, 50, 100, and 200 mL ingestion, respectively; B25, B50, B100, and B200, beer of 25, 50, 100, and 200 mL ingestion, respectively. * P < 0.05 vs. each baseline.

### Effects of Ingesting Beer on Hemodynamic Parameters

Baseline HR and BP did not significantly differ. Although HR in B25, B50, and B100 tended to increase, the values did not reach statistical significance. HR in B200 significantly increased at 15 min post-ingestion (ES = 0.76), but not in any AF trial. Systolic BP, diastolic BP, and mean BP did not significantly change throughout the experimental period, but post-ingestion diastolic BP slightly decreased in the B50, B100, and B200 trials (Table 2).

**TABLE 2:** Changes in hemodynamic parameters.

**Variables**	**Baseline**	**5 min**	**15 min**	**30 min**	**60 min**	**Trial**	**Time**	**Interaction**
Heart rate, beats/min								
AF25	604	573	543	553	563	*F* = 0.485	*F* = 1.521	*F* = 2.298
AF50	573	563	583	593	573			
AF100	571	561	562	572	562	*P* = 0.842	*P* = 0.197	*P* < 0.001
AF200	613	593	603	573	573			
B25	582	572	562	552	593			
B50	593	594	635	624	583			
B100	583	573	613	613	583			
B200	563	634*	675*	645*	614*			
Systolic BP, mmHg								
AF25	1244	1213	1223	1223	1243	*F* = 0.075	*F* = 0.835	*F* = 1.356
AF50	1213	1213	1203	1253	1233			
AF100	1244	1254	1234	1243	1233	*P* = 0.999	*P* = 0.504	*P* = 0.115
AF200	1233	1212	1183	1254	1202			
B25	1223	1223	1244	1233	1222			
B50	1224	1213	1253	1234	1193			
B100	1245	1265	1243	1234	1225			
B200	1225	1224	1204	1224	1253			
Diastolic BP, mmHg								
AF25	692	702	682	682	692	*F* = 0.590	*F* = 2.271	*F* = 0.788
AF50	701	711	711	711	721			
AF100	701	701	721	701	691	*P* = 0.762	*P* = 0.062	*P* = 0.771
AF200	711	712	712	722	712			
B25	702	721	702	712	732			
B50	722	712	691	691	702			
B100	712	743	712	722	713			
B200	682	711	662	682	693			
Mean BP, mmHg								
AF25	892	902	882	872	912	*F* = 0.097	*F* = 1.648	*F* = 0.915
AF50	872	892	902	911	902			
AF100	893	892	913	892	892	*P* = 0.998	*P* = 0.163	*P* = 0.583
AF200	902	892	892	912	912			
B25	892	902	892	903	912			
B50	902	912	902	892	902			
B100	872	892	882	892	902			
B200	893	892	892	893	891			

Data are expressed as mean ± SE. BP, blood pressure; AF25, AF50, AF100, and AF200, alcohol-free beer of 25, 50, 100, and 200 mL ingestion, respectively; B25, B50, B100, and B200, beer of 25, 50, 100, and 200 mL ingestion, respectively. * P < 0.05 vs. each baseline.

### Relationships Between Alcohol Levels and Arterial Stiffness

In all pooled data of main experiments in beer trials from 15 min to 60 min, estimated circulating alcohol levels were significantly and negatively correlated with changes in hbPWV (*r* = −0.47, *P* < 0.001), baPWV (*r* = −0.31, *P* < 0.01), haPWV (*r* = −0.45, *P* < 0.001), and CAVI (*r* = −0.60, *P* < 0.001). Statistical correlations tended to be stronger between circulating alcohol and hbPWV, haPWV, and CAVI than baPWV. However, although the same volume of beer was consumed, changes in arterial stiffness did not correlate significantly with the volume of beer consumed per body mass (hbPWV 15 min, *r* = 0.33, *P* = 0.39; baPWV 15 min, *r* = −0.08, *P* = 0.84; haPWV 15 min, *r* = 0.04, *P* = 0.93; CAVI 15 min, *r* = 0.06, *P* = 0.88).

### Supplementary Experimental Findings

**FIGURE 3:**
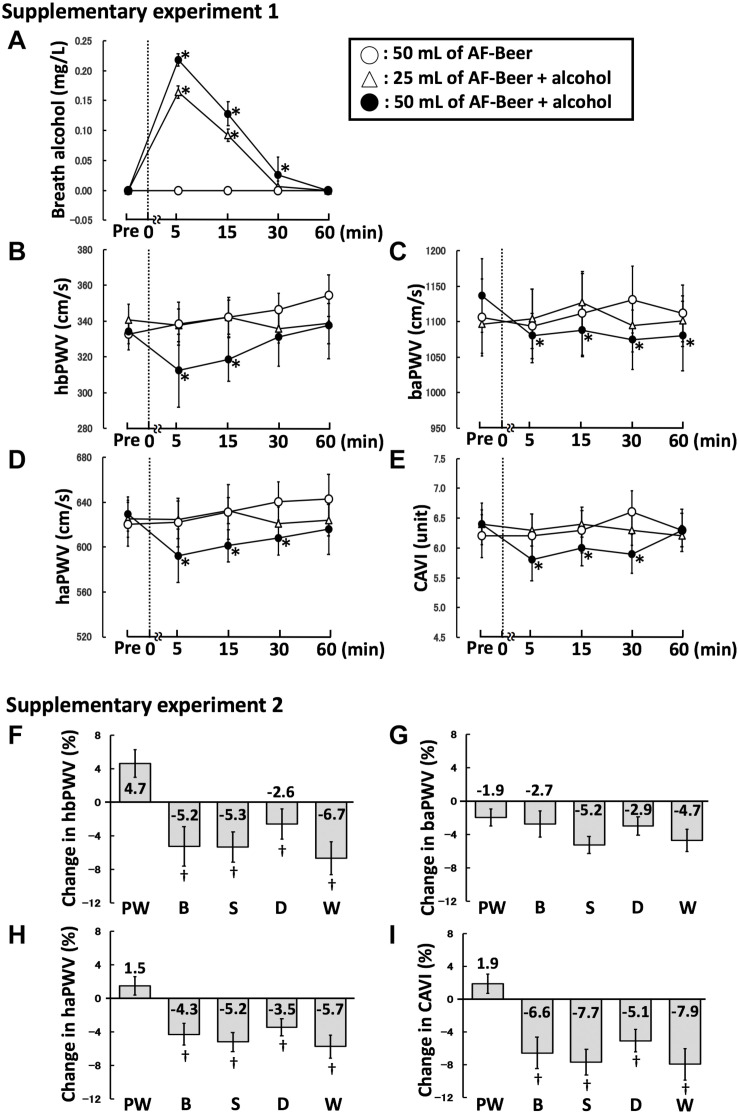
Supplementary experimental findings. Changes in breath alcohol levels **(A)**, hbPWV **(B)**, baPWV **(C)**, haPWV **(D)**, and CAVI **(E)** in Supplementary Experiment 1. Comparisons of the changes in hbPWV **(F)**, baPWV **(G)**, haPWV **(H)**, and CAVI **(I)** in Supplementary Experiment 2. Broken lines, time points of beverage ingestion; Pre, baseline, AF-Beer, alcohol-free beer; AF-Beer + alcohol, alcohol mixed with alcohol-free beer as positive control; PW, pure water; B, beer; S, sake; D, Japanese distilled spirits; W, whisky; hbPWV, heart-brachial pulse wave velocity; baPWV, brachial-ankle pulse wave velocity; haPWV, heart-ankle pulse wave velocity; CAVI, cardio-ankle vascular index; *p < 0.05 vs. each pre; ^†^p < 0.05 vs. PW. Data are expressed as mean ± SE.

Post-ingestion PWVs and CAVI in the AF50 trial (Supplementary Experiment 1) became significantly reduced when the participants consumed 50 mL of alcohol mixed with alcohol-free beer (AF + Alc50 trial; hbPWV 15 min, ES = 1.00; baPWV 15 min, ES = 0.74; haPWV 15 min, ES = 0.87; CAVI 15 min, ES = 0.73). However, PWV and CAVI did not change significantly in the AF + Alc25 trial (hbPWV 15 min, ES = 0.04; baPWV 15 min, ES = 0.64; haPWV 15 min, ES = 0.03; CAVI 15 min, ES = 0.00). These findings supported those of the B25 and B50 trials (Figures 3A–E).

Pulse wave velocity and CAVI were significantly reduced after ingesting 100 mL of B, S, D, and W but not PW (Supplementary Experiment 2). In addition, reductions in hbPWV, haPWV, and CAVI at 15 min post-ingestion were significantly greater for B (hbPWV, ES = 1.64; haPWV, ES = 1.19; CAVI, ES = 1.49), S (hbPWV, ES = 1.52; haPWV, ES = 1.43; CAVI, ES = 1.83), D (hbPWV, ES = 1.10; haPWV, ES = 1.30; CAVI, ES = 1.72), and W (hbPWV, ES = 2.01; haPWV, ES = 1.62; CAVI, ES = 1.66) than for PW. These degrees of reduction did not significantly differ among trials of alcohol, regardless of type. However, the reductions in baPWV were small, and did not significantly differ across trial (B vs. PW, ES = 0.20; S vs. PW, ES = 0.84; D vs. PW, ES = 0.23; W vs. PW, ES = 0.55) (Figures 3F–I).

## Discussion

The salient findings are as follows. The CAVI and PWVs were significantly reduced in B50, B100, and B200 trials at 30–60 min post-ingestion, but not in either B25 or all AF trials. The findings of the supplementary experiments supported the results of main experiments, indicating that the alcohol in beer was the main contributor to acute reduction in PWV and CAVI. To the best of our knowledge, this is the first study to clarify the dose of beer required to induce reductions in arterial stiffness.

A significant reduction in arterial stiffness has been determined by ingesting ∼ 200 mL of beer, which corresponds to mild-to-moderate consumption (Nishiwaki et al., 2017a). However, to our knowledge, the amount of beer required to induce an acute reduction in arterial stiffness has remained unknown. The present results showed that CAVI and PWVs started to fall in the B50 trial and were reduced in the B100 and B200 trials, but not in the B25 and all AF trials. The positive control in supplementary experiment confirmed these findings of PWVs and CAVI. The gold-standard for assessing arterial stiffness is generally PWVs (Tanaka and Safar, 2005; Vlachopoulos et al., 2010; Ohkuma et al., 2017), and CAVI is also an index of arterial stiffness from the aorta to the ankle, after adjustment for BP, which is a major confounding factor (Shirai et al., 2011; Matsushita et al., 2019). In general, haPWV and CAVI are systemic indices of arterial stiffness from the aorta to the ankle (Tomiyama et al., 2003; Shirai et al., 2011). They comprise hbPWV that mainly reflects arterial stiffness of the upper limbs from the aorta to the brachial, which can serve as a marker of arterial stiffening of the proximal aorta (Sugawara et al., 2018, 2019), and baPWV, which mainly reflects arterial stiffness of central and lower limbs from the level of the brachial (thoracoabdominal) to the ankle (Yamashina et al., 2002). In addition, post-ingestion BP did not significantly change in either the B50, B100, or B200 trials. Although HR influences on PWV and HR significantly increased in the B200 trial (Callaghan et al., 1984; Lantelme et al., 2002), previous studies have indicates that fluctuations in HR between 60 and 70 beats/min do not obviously affect PWV values (Callaghan et al., 1984; Lantelme et al., 2002). Thus, our findings indicate that acute beer ingestion of about 50 mL (0.77 ± 0.05 mL of beer/kg body mass and 0.034 ± 0.002 g of alcohol/kg body mass) begins to reduce arterial stiffness.

The main constituents of beer are alcohol, antioxidant substances, water, and sugar (Arranz et al., 2012; Karatzi et al., 2013; de Gaetano et al., 2016). We previously showed that antioxidant substances and sugar do not affect beer-induced acute reduction in arterial stiffness (Nishiwaki et al., 2017a). The present study identified significant and negative correlations between estimated circulating levels of alcohol and changes in arterial stiffness. Furthermore, the findings of the two supplementary experiments supported the main results of present study, namely, that arterial stiffness was significantly reduced regardless of the type of alcohol consumed. Therefore, these findings show that the alcohol in beer is the main contributor to the acute reduction in arterial stiffness associated with ingesting a small amount of beer.

The physiological mechanism underlying vascular responses to alcohol ingestion have not yet been elucidated. However, changes in arterial stiffness are generally thought to result from structural changes in elastin and collagen content, functional changes in vasoconstrictor tone and endothelial functions, or a combination of both (Tanaka and Safar, 2005). Because the structure of the arterial walls is believed to change over weeks or years (Tanaka et al., 2000; Tanaka and Safar, 2005; Maeda et al., 2009; Sugawara et al., 2009), an acute change in arterial stiffness is probably mediated by functional changes (Sugawara et al., 2004; Maeda et al., 2009). Acute alcohol ingestion alters BP variability, which is index of sympathetic control of vasomotor tone (Buckman et al., 2015). Thus, one possible mechanism underlying acute effects of beer or alcohol ingestion on arterial function may be related to the change in sympathetic control of vasomotor tone. In addition, recent studies indicate that mental stress or comic movies-induced mirthful laughter affects arterial stiffness and/or vascular function (Vlachopoulos et al., 2006; Sugawara et al., 2010). Thus, ingesting a small amount of beer or alcohol might change mood states of participants, and thereby reducing vasomotor tone or arterial stiffness. Alternatively, studies *in vitro* have demonstrated that a low concentration of alcohol can promote NO release from endothelium by upregulation of NO synthase (Toda and Ayajiki, 2010; Zhou et al., 2016). NO is an important endogenous vasoactive substance that plays a key role in regulating blood pressure and protecting against pathological vascular damage. Therefore, ingesting alcohol might increase NO bioavailability in the vascular endothelium, thus reducing arterial stiffness (Karatzi et al., 2013). However, in this study, there is a lack of direct data to support our view and further studies are required.

Recent systematic reviews and meta-analyses found that an increase in PWV of 1 m/s corresponds to a >10% increase in risk for cardiovascular events or mortality (Vlachopoulos et al., 2010), and thus a reduction in arterial stiffness is considered of paramount importance. Many previous studies have demonstrated that both acute and chronic vascular responses to exercise are probably relevant (Kingwell et al., 1997; Kakiyama et al., 2005; Nishiwaki et al., 2015; Yamato et al., 2016). Thus, our results raise the possibility that repeated acute reductions in arterial stiffness and its accumulation would induce persistent reductions in arterial stiffness. Furthermore, according to our previous studies (Nishiwaki et al., 2014), we applied the linear regression between age and CAVI, and estimated “vascular age.” In the present study, alternations of vascular age corresponded to changes in 5–9 years between baseline and during beer ingesting trials. Therefore, our findings indicate that the magnitudes of reduction are not only statistically significant, but also clinically meaningful. However, as of now, whether regular ingestion of a small amount of beer or alcohol in daily life reduces arterial stiffness are unclear, and further intervention studies are thus needed.

Recent combined analysis by large-scale prospective studies have demonstrated that the threshold for lowest risk of total cardiovascular diseases and all-cause mortality is about 100 g of alcohol/week (Wood et al., 2018), which partially supports J-shaped relationships (Di Castelnuovo et al., 2006; Costanzo et al., 2011; Arranz et al., 2012; Poli et al., 2013; de Gaetano et al., 2016; Zhou et al., 2016; Panagiotakos et al., 2019). In addition, some meta-analyses have indicated that relative risks of vascular events can be reduced at 2–3 g/day of alcohol consumption and significantly reduced at approximately 5 g/day (Di Castelnuovo et al., 2006; Costanzo et al., 2011; Poli et al., 2013). In the present study, 50 and 100 mL of beer contained 2.2 and 4.4 g of alcohol, respectively. Therefore, our experimental findings can have high validity, because they are in line with these epidemiological results. However, the J-shaped association is not widely accepted, especially in terms of relationships between alcohol intake and stroke, cancer, and liver diseases (Arranz et al., 2012; Poli et al., 2013; de Gaetano et al., 2016; Zhou et al., 2016; Wood et al., 2018). Indeed, the American Heart Association states that those who consume excessive amounts of alcohol as well as those who abstain from alcohol, should not be encouraged to drink alcohol for health reasons (Arranz et al., 2012; Arnett et al., 2019). Thus, whether consuming a small amount (50–100 mL) of beer actually affects arterial stiffness, total health, and longevity remains unresolved and further investigations are required.

The strengths of our study include two supplementary experiments of positive control and ingesting different types of alcoholic beverages. However, this study has several important potential limitations. First, experimental studies have measured absolute amounts and body mass-adjusted relative amounts to determine alcohol intake (Krnic et al., 2011; Karatzi et al., 2013; Fantin et al., 2016; Nishiwaki et al., 2017a). Because recommended alcohol intake is defined by absolute amounts, and we intended that our study should be easily generalizable to the clinical settings, participants were given four different small absolute amounts of beer (25, 50, 100, and 200 mL). However, changes in arterial stiffness did not significantly correlate with relative amounts of alcohol per kilogram of body mass. Thus, the required dose for reduction in arterial stiffness was not profoundly affected by the minor differences in mass-based relative amounts ingested by each participant. Second, individual physiological responses to alcohol ingestion are affected by factors such as age, sex, amounts habitually consumed, and the arterial stiffness. Thus, our results are specific to young, healthy, male, mild-to-moderate consumers of alcohol, although it seems unlikely that changes in arterial stiffness can be profoundly affected by ingesting small amounts of beer. The number of participants was also very small although several supplementary experiments were performed. Therefore, statistical interpretation is limited and additional investigations using different protocols and study populations might uncover important new insights into the relationship between arterial stiffness and alcohol ingestion.

In conclusion, we found that about 50 mL of ingested beer (0.77 ± 0.05 mL of beer/kg body mass and 0.034 ± 0.002 g of alcohol/kg body mass) begins to reduce arterial stiffness, and that the reduction in arterial stiffness is mainly attributable to the alcohol in beer. These findings could thus offer new insights into the development of better methods for preventing arterial stiffening, regulating the circulation system, and vascular biology, especially from the viewpoint of blood distribution during physiological tasks or alcohol ingestion.

## Data Availability Statement

All datasets generated for this study are included in the article/Supplementary Material.

## Ethics Statement

The studies involving human participants were reviewed and approved by the Human Ethics Committee at the Osaka Institute of Technology (approval numbers: 2016-5 and 2017-55). The patients/participants provided their written informed consent to participate in this study.

## Author Contributions

MN, TY, and RN conceived, designed, and performed the study and analyzed the data. MN, TY, RN, and NM wrote the manuscript. MN and NM interpreted the data. All authors approved the final version of the manuscript.

## Conflict of Interest

The authors declare that the research was conducted in the absence of any commercial or financial relationships that could be construed as a potential conflict of interest.
