# Supplementary material for: Dose of Alcohol From Beer Required for Acute Reduction in Arterial Stiffness
Source: Front Physiol. 2020 Aug 28;11:1033. doi: 10.3389/fphys.2020.01033 (PMC7485316; doi:10.3389/fphys.2020.01033)
Supplement: Supplementary file 1 [file Table_1.DOCX]

Additional information 1. Changes in hemodynamic parameters in Supplementary experiment 1

Data are expressed as mean ± SE. BP, blood pressure; AF-Beer, Alcohol-free beer.
