# Supplementary material for: Dose of Alcohol From Beer Required for Acute Reduction in Arterial Stiffness
Source: Front Physiol. 2020 Aug 28;11:1033. doi: 10.3389/fphys.2020.01033 (PMC7485316; doi:10.3389/fphys.2020.01033)
Supplement: Supplementary file 3 [file Table_3.DOCX]

Additional information 3. Changes in hemodynamic parameters in Supplementary experiment 2

Data are expressed as mean ± SE. BP, blood pressure; PW, pure water; B, beer; S, sake; D, Japanese distilled spirits; W, whisky.
